# Metabolites derived from fungi and bacteria suppress in vitro growth of *Gnomoniopsis smithogilvyi*, a major threat to the global chestnut industry

**DOI:** 10.1007/s11306-022-01933-4

**Published:** 2022-09-15

**Authors:** Matias Silva-Campos, Damien L. Callahan, David M. Cahill

**Affiliations:** 1 School of Life and Environmental Sciences, Deakin University, Geelong Waurn Ponds Campus, Geelong, VIC 3216 Australia; 2 School of Life and Environmental Sciences, Centre for Cellular and Molecular Biology, Deakin University, Burwood Campus, Burwood, VIC 3125 Australia

**Keywords:** *Gnomoniopsis smithogilvyi*, Biological control, nVOCs, VOCs, *Trichoderma* spp., *Bacillus subtilis*

## Abstract

**Introduction:**

Chestnut rot caused by the fungus *Gnomoniopsis smithogilvyi* is a disease present in the world’s major chestnut growing regions. The disease is considered a significant threat to the global production of nuts from the sweet chestnut (*Castanea sativa*)*.* Conventional fungicides provide some control, but little is known about the potential of biological control agents (BCAs) as alternatives to manage the disease.

**Objective:**

Evaluate whether formulated BCAs and their secreted metabolites inhibit the in vitro growth of *G. smithogilvyi.*

**Methods:**

The antifungal potential of BCAs was assessed against the pathogen through an inverted plate assay for volatile compounds (VOCs), a diffusion assay for non-volatile compounds (nVOCs) and in dual culture. Methanolic extracts of nVOCs from the solid medium were further evaluated for their effect on conidia germination and were screened through an LC–MS-based approach for antifungal metabolites.

**Results:**

Isolates of *Trichoderma* spp., derived from the BCAs, significantly suppressed the pathogen through the production of VOCs and nVOCs. The BCA from which *Bacillus subtilis* was isolated was more effective in growth inhibition through the production of nVOCs. The LC–MS based metabolomics on the nVOCs derived from the BCAs showed the presence of several antifungal compounds.

**Conclusion:**

The results show that *G. smithogilvyi* can be effectively controlled by the BCAs tested and that their use may provide a more ecological alternative for managing chestnut rot. The in vitro analysis should now be expanded to the field to assess the effectiveness of these alternatives for chestnut rot management.

**Supplementary Information:**

The online version contains supplementary material available at 10.1007/s11306-022-01933-4.

## Introduction

One of the main threats to the production of nuts from sweet chestnut (*Castanea sativa* Mill.) worldwide is the diseases caused by the fungus *Gnomoniopsis smithogilvyi* (Ascomycete, Gnomoniaceae, Diaporthales). Incursions of the pathogen have been reported in several countries, including Australia (Shuttleworth et al., 2012), Chile (Cisterna-Oyarce et al., 2022), India (Dar and Rai, 2015), Spain (Trapiello et al., 2018), Italy (Visentin et al., 2012) and Switzerland (Dennert et al., 2015), among others. Under the right conditions of humidity and temperature, the pathogen colonises the starch-rich nut endosperm affecting its integrity and colour, which makes it unmarketable (Shuttleworth and Guest, 2017). Currently, in conventional farming, alternatives to growers for managing the disease include the application of fungicides pre-harvest (Australian Pesticides & Veterinary Medicines Authority, 2021; Chestnuts Australia, 2018) and sanitization of nuts with disinfectant post-harvest (Chestnuts Australia, 2017). However, these measures seem insufficient to control the incidence of *G. smithogilvyi* as disease peaks are still of considerable magnitude. For example, studies have found levels of nut infection of 91% in Switzerland (Dennert et al., 2015), 93.5% in Italy (Lione et al., 2015), and 70% in Australia (Shuttleworth et al., 2013). This demonstrates the significant risk to which chestnut production worldwide is exposed due to *G. smithogilvyi*. Therefore, there is an urgent need for the application of new strategies that could complement or replace those used currently to control the pathogen.

Biological control agents (BCAs) such as fungi and bacteria have emerged as an effective and environmentally friendly alternative to control several plant diseases (Minchev et al., 2021; Syed-Ab-Rahman et al., 2018). Within the vast microbial world, bacteria and fungi from the genus *Bacillus*, *Pseudomonas*, and *Trichoderma,* for example, have been examined for their production of volatile and non-volatile metabolites that show antimicrobial activity. For example, volatile organic compounds (VOCs) emitted by *Bacillus subtilis*, *Pseudomonas putida* and *Trichoderma harzianum* effectively suppressed the in vitro mycelial growth of *Alternaria solani* (Zhang et al., 2020), *Colletotrichum gloeosporides* (Sheoran et al., 2015) and *Fusarium oxysporum* (Li et al., 2018), respectively. Similarly, non-volatile compounds (nVOCs) secreted by *B. subtilis*, *P. putida* and *T. harzianum* showed significant growth inhibition of *F. graminearum* (Munakata et al., 2022), *Colletotrichum acutatum* (Moreira et al., 2014), and *Magnaporthe oryzae* (Zhao et al., 2020). However, there have been very limited studies on whether such microbially derived metabolites are active against *G. smithogilvyi.*

We examined several BCA formulations for their effectiveness in suppressing *G. smithogilvyi* growth. These formulations included species of the genera *Bacillus*, *Pseudomonas*, and *Trichoderma*. We examined the effectiveness of VOCs and nVOCs derived from the BCAs in suppressing pathogen growth in vitro. Through high-resolution liquid chromatography-mass spectrometry (LC–MS), a comparative study of the composition of the nVOCs produced by the BCAs was undertaken. There were clear differences in the metabolomes produced by the BCAs and which was closely linked to the differential activity observed. A new avenue for disease management that is more environmentally friendly and sustainable for the complex chestnut rot disease has thus been provided.

## Materials and methods

### *G. smithogilvyi* isolates and BCAs identification

*Gnomoniopsis smithogilvyi* isolates, B15 and F1N1, were obtained from rotted nut tissues cultured onto potato dextrose agar (PDA, DifcoTM, New Jersey, USA). The BCAs (TRI, SUP and D25) used in this study correspond to commercially available formulations composed of a mixture of microorganisms including *Trichoderma* spp., *Bacillus subtilis*, and *Pseudomonas putida*. For molecular identification of the *G. smithogilvyi* and the fungi or bacteria in two (TRI, SUP) of the BCAs DNA was extracted from 5-day-old colonies grown on PDA at 23 °C using a commercial kit (Quick-DNA™ Fungal/Bacterial Miniprep Kit, Zymo Research) following the manufacturer’s instructions. For *G. smithogilvyi* the Internal Transcribed Spacer (ITS) region was amplified with primers ITS-5 (forward) and ITS-4 (reverse) according to White et al. (1990). Microbial profiling of BCAs was carried out by amplifying the ITS (fungi), 16S (bacteria) regions, and Sanger sequencing was performed by the Australian Genome Research Facility, Melbourne, Australia. All sequences were analysed using the NCBI-GenBank database.

### BCAs preparation for analysis of VOCs and nVOCs

The BCAs were dissolved in sterile water (stH_2_O) to make a stock solution of 20 mg/mL or 20 μL/mL depending on the formulation format (Supplementary Table 1). The stock solution was activated for 4 h at 23 °C in the dark, filtered and a range of tenfold dilutions were made from 20 to 0.002 mg/mL (TRI, SUP) or 20 to 0.002 μL/mL (D25).

### Effect of nVOCs on *G. smithogilvyi* growth in vitro

**Fig. 1:**
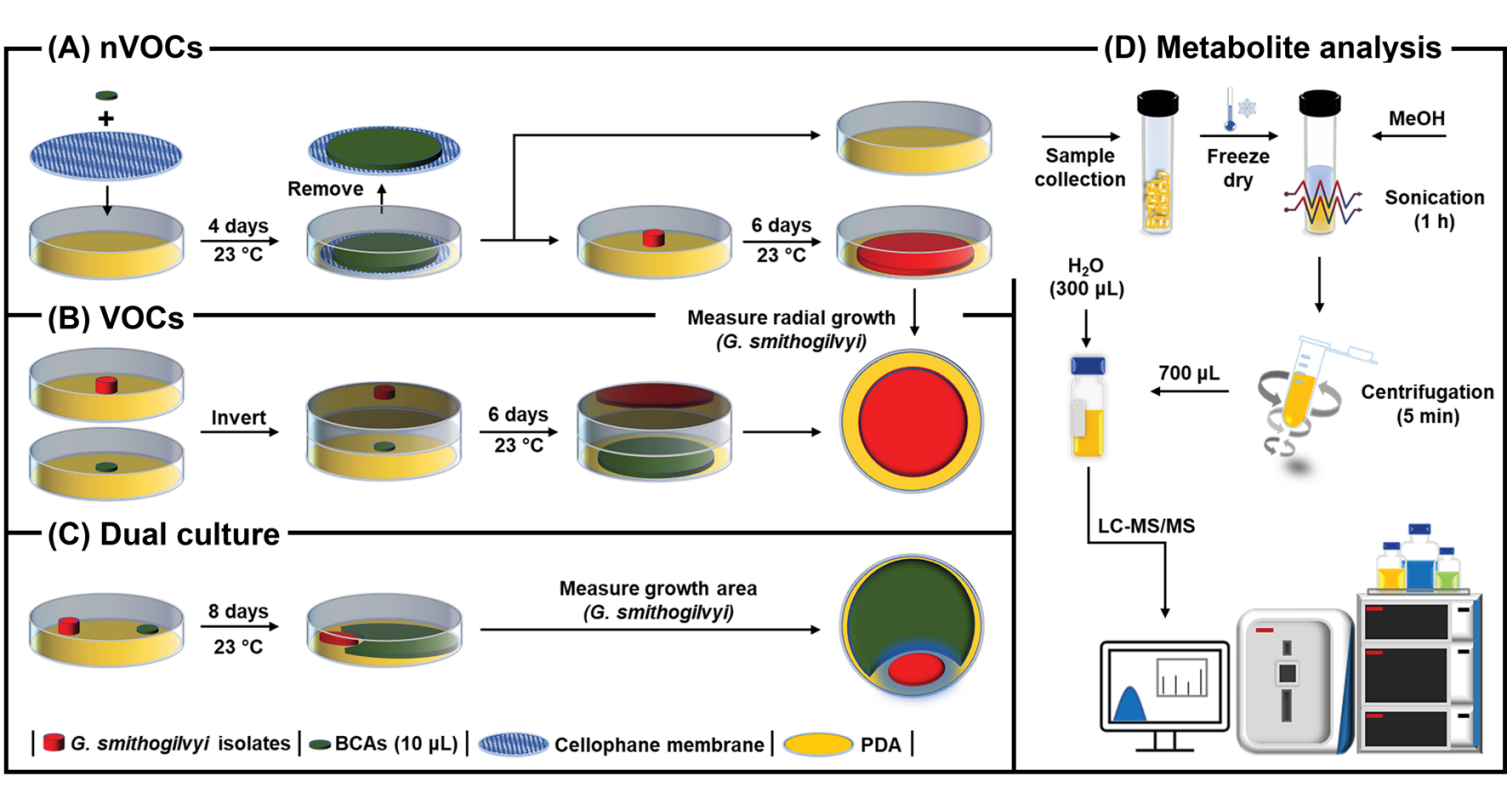
Schematic representation of the experimental procedures performed to evaluate the biological control agents against *G. smithogilvyi*. **A** Non-volatile compounds assay (nVOCs). See Sect. 2.3, **B** Volatile compounds (VOCs) assay. See Sect. 2.4, **C** Dual culture assay. See Sect. 2.5, **D** Extraction and analysis of metabolites through LC–MS. See Sect. 2.7

The effect of nVOCs derived from the cultured BCAs on *G. smithogilvyi* mycelial growth was evaluated following the procedure described by Steyaert et al. (2016) with some modifications (Fig. 1A). Firstly, sterile cellophane membrane discs (90 mm-in-diameter) were overlain on PDA using forceps. Then, 10 μL of each BCA concentration or stH_2_O for the control treatment were placed on a 5 mm-in-diameter sterile filter paper disc (no.1, Whatman®, Cytiva, UK) in the middle of the plate. Plates were sealed with parafilm and incubated at 23 °C in the dark for four days. After incubation, the cellophane membrane was removed carefully to ensure that BCA tissue did not contact the medium. Plates were then inoculated in the centre with a 6 mm-in-diameter plug of a five-day-old culture of *G. smithogilvyi* isolate B15 or F1N1. Plates were then sealed with Parafilm® (Amcor, Switzerland) and incubated at 23 °C in the dark for 6 days. Mycelial growth of the isolates was measured radially from the plug border to the edge of the colony by taking three measurements perpendicular to each other. The experiment was carried out with three replicates for each BCA × isolate × concentration combination and repeated 3 times.

### Effect of VOCs on *G. smithogilvyi* growth in vitro

The effect of VOCs on *G. smithogilvyi* mycelial growth was evaluated through an inverted Petri plate technique described by Dennis and Webster (1971) with some modifications (Fig. 1B). BCAs (10 μL) at each concentration or stH_2_O for control treatments were placed on a 5 mm-in-diameter sterile filter paper disc (Whatman No. 1) in the centre of the PDA plate. A second PDA plate was inoculated with a 6 mm-in-diameter plug from a five-day-old *G. smithogilvyi* culture of either isolate B15 or F1N1. The plate containing the pathogen was mounted facing downward on top of the plate containing the BCA or stH_2_O and then the two plates sealed with two layers of Parafilm®. Finally, plates were incubated at 23 °C in the dark for 6 days. After incubation, mycelial growth was measured as described above. The experiment was carried out with three replicates for each BCA × isolate × concentration combination and repeated 3 times.

### Effect of BCAs on *G. smithogilvyi* growth in a dual culture assay

On one side of a PDA Petri dish (90 mm) a 6 mm-in-diameter agar plug of a five-day-old culture of *G. smithogilvyi* was placed 1 cm from the edge (Fig. 1C). On the opposite side, a 5 mm-in-diameter sterile filter paper disc that contained 10 μL of each BCA prepared at 20 mg/mL (TRI, SUP), 20 μL/mL (D25) and stH_2_O for the control treatment was also placed 1 cm from the edge. Plates were then sealed with parafilm and incubated at 23 °C in the dark. Growth area of each isolate was measured following 6 days of incubation and then again at 8 days. Growth area of each isolate was determined using a free software program (ImageJ, version 1.51j8, U.S. National Institutes of Health, USA). The experiment was carried out with three replicates and repeated 3 times.

### Extraction of nVOCs from solid medium

The BCAs were grown on PDA media overlayed with cellophane membrane at 23 °C for 4 days. Then, the cellophane membrane containing BCAs was removed, and 5 g/plate of the medium was used for nVOCs extraction (Fig. 1D). The agar medium sample was placed in 10 mL capped culture tubes, frozen with liquid nitrogen for 4 min, and then freeze dried (VirTis BenchTop Pro freeze dryer, SP scientific, USA) for 48 h. The instrument was set at − 60 °C with a vacuum pressure of 200 m Torr. Freeze-dried samples were resuspended and adjusted to 50 mg/mL with 100% methanol (HPLC grade, Merck, Germany). The solution was then subjected to ultrasonication (Power sonic 410, Hwashin technology, South Korea) set at 30 °C for 60 min. Then, 1.5 mL of the solution was transferred to a 2 mL centrifuge tube to pellet undissolved particles by centrifugation at 11,000 rpm for 5 min. Finally, 300 μL of deionised water was added to 700 μL of the supernatant and kept in 2 mL glass vials at − 20 °C until analysis by LC–MS. Five plates treated with BCAs or stH_2_O were used as technical replicates for analysis.

### High-performance liquid chromatography fractionation of nVOCs

Samples were fractionated using a high-performance liquid chromatography (HPLC) system with a diode array detector (UltiMate™ 3000 BioRS, ThermoFisher Scientific, USA). The LC parameters were as follows: column 4.6 × 150 mm, 3 µm Luna C18 (2) (Phenomenex, USA), column temperature 25 °C, flow rate 0.75 mL/min, with gradient elution. Mobile phase A was 0.1% formic acid in water, mobile phase B was 0.1% formic acid in acetonitrile (LC–MS grade solvents used). The initial mobile phase composition was 5% B which linearly increased to 100% B over 14 min with a 3-min hold at 100% B then re-equilibration for 5 min at 5% B, giving a total run time of 22 min. The diode array detector was set from 220 to 800 nm. Sample injection volume was 25 µL and fractions were collected every 1 min from 1 to 16 min.

### Analysis of nVOCs from solid media and HPLC fractions

Samples were analyzed using an ultra-high resolution mass spectrometer coupled with liquid chromatography (LC–MS. Vanquish Flex UHPLC coupled with an OrbitrapExploris-240, ThermoFisher Scientific). The LC parameters were as follows: column 2.1 × 100 mm, 1.8 µm C18 Zorbax Elipse plus (Agilent, USA), column temperature 30 °C, flow rate 0.4 mL/min, with gradient elution. Mobile phase A was 0.1% formic acid in water, mobile phase B was 0.1% formic acid in acetonitrile, LC–MS grade solvents were used. The initial mobile phase composition was 5% B which held for 1 min then linearly increased to 100% B over 9 min with a 2 min hold at 100% B then re-equilibration for 3 min at 2% B, giving a total run time of 15 min. The H-ESI source settings were: ion spray voltage 3800 V, sheath gas 50 (arb. units), sweep gas 1 arb, ion transfer tube 325 °C, vaporizer temperature 350 °C. The MS was operated in data dependant MS/MS mode with a full scan between 90 and 1350 m/z at a resolution of 120,000 and MS/MS scans with a resolution of 15,000. The cycle time was 0.6 cycles per second (1 full scan 7 MS/MS scans per 0.6 s cycle). Three normalised collision energies were used for MS/MS (20, 40, 60 NCE) and the MS/MS threshold was 5000 counts. The injection volume of sample extracts was 5 µL. The Easy-IC internal calibration was used giving sub-1 ppm mass accuracy. Data was collected in both positive and negative ionization mode in separate runs.

### Annotation of nVOCs detected by LC–MS

Annotation, chromatographic deconvolution, library searching, and data visualization of putative compounds was performed with the software program Compound Discoverer 3.3 (ThermoFisher Scientific). Compounds were annotated based on comparing their calculated elemental composition against that of 220 compounds from a manually constructed library of compounds reported for *Bacillus*, *Pseudomonas* and *Trichoderma* species in the literature. Additionally, compounds were compared against the ChemSpider database. Elucidation of putative compound structures was carried out by applying the software-integrated function Fragment Ion Search (FISh) scoring algorithm which matches fragments ions to the theoretical structure of the parent molecule (Wang et al., 2019; Welling et al., 2021).

### Antifungal activity of crude extracts against *G. smithogilvyi*

The fungicidal activity of methanolic crude extracts of the BCAs was evaluated against *G. smithogilvyi* isolate B15 by means of a disc diffusion assay on PDA. Firstly, the methanolic nVOCs extract was concentrated by evaporating the solvent in a vacuum concentrator (model RVC 2–18 CDplus, Christ, Germany) set a 35 °C for 2 h and adjusted to 500 mg/mL with 70% methanol in water. Then, 150 μL of a conidial suspension (1.7 × 10^6^ conidia/mL) of isolate B15 was dispersed on the surface of the medium in each Petri plate (90 mm). Filter paper discs (5 mm-in-diameter) were equidistantly placed onto the medium and then inoculated with 10 μL of the crude extract or 70% methanol in water as the control. Plates were incubated at 23 °C in the dark for 3 days and the diameter of the zone of inhibition was measured across two sections perpendicular to each other. The experiment was performed in quadruplicate and repeated twice.

### Evaluation of antifungal activity of HPLC fractions

The collected fractions were evaluated for their potential to inhibit *G. smithogilvyi* isolate B15 mycelial growth on PDA. Fractions were vacuum evaporated as described above and resuspended in 100 μL of 70% methanol in water. Then, 30 μL of each fraction or 70% methanol in water for the control were evenly dispersed using a sterile L-shaped spreader on each of three individual PDA plates (60 mm-in-diameter). A plug of 6 mm-in-diameter from a five-day-old *G. smithogilvyi* isolate B15 culture was then placed on the agar surface in the centre of the plate. Plates were incubated at 23 °C in the dark for 3 days and the colony area was measured using ImageJ software.

### Statistical analysis and data visualisation

Statistical analysis and graphical visualisation were performed using a commercial software program (GraphPad Prism, vers. 8.0.0 for Windows, GraphPad Software, USA). The effect of the VOCs, nVOCs and dual culture on mycelial growth of both *G. smithogilvyi* isolates were evaluated by analysis of variance (two-way ANOVA). The mean of each treatment was subjected to multiple comparisons with Dunnett’s post-hoc test at a 5% significance level (*p* = 0.05). Disc diffusion assay results were compared with one-way ANOVA with the same post-hoc test. UpSet plots were used to show distribution and correlation of unique and shared metabolites between BCAs and were constructed with Python (Lex et al., 2014).

## Results

### Effect of nVOCs on *G. smithogilvyi* mycelial growth

**Fig. 2:**
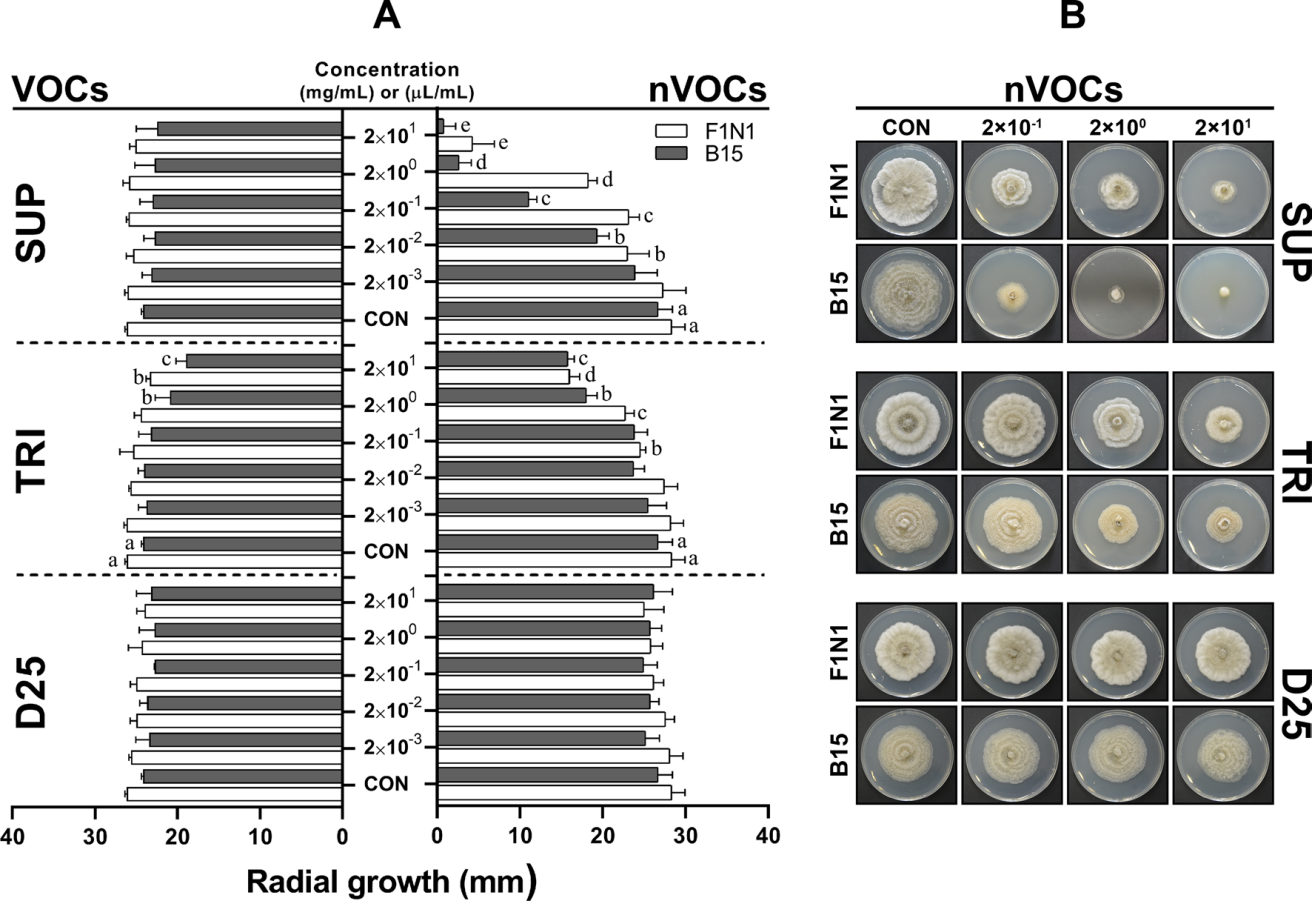
Effect of volatile organic compounds (VOCs) and non-volatile compounds (nVOCs) on mycelial growth of *G. smithogilvyi* isolates B15 and F1N1. **A** Shows the effect of VOCs (Left) and nVOCs (right) on the radial growth of the isolates at each concentration tested (centre). **B** Representation of the effect of nVOCs secreted by the highest three BCAs concentrations on the mycelial growth of both isolates. Plates were incubated at 23 °C in the dark for six days. Means ± SEM labelled with the same letter are not significantly different to the control according to Dunnett’s test at *p* = 0.05

The antifungal properties of the nVOCs secreted by BCAs on PDA medium differed greatly in their effectiveness in reducing *G. smithogilvyi* mycelial growth (Fig. 2A, B). The results showed that the nVOCs exuded by SUP were comparatively more effective in reducing the mycelial growth of both isolates than of TRI and D25. There were significant differences (*p* < 0.05) in growth compared to the control treatment at concentrations of SUP that ranged between 2 × 10^–2^ and 2 × 10^1^ (mg/mL). Treatment with the highest concentration resulted in the minimum radial growth for both isolates, with F1N1 reaching 4.27 ± 1.5 mm and B15 0.81 ± 0.81 mm compared with the control of 28.36 ± 1.6 mm (F1N1) and 26.68 ± 1.7 mm (B15). The second most effective nVOCs that inhibited mycelial growth were produced by TRI. Significant differences (*p* < 0.05) were found between the control and treatments at concentrations ranging from 2 × 10^–1^ to 2 × 10^1^ (mg/mL). Again, at the highest concentration of TRI, both isolates were restricted to growth of 16.0 ± 0.7 mm (F1N1) and 15.8 ± 0.4 mm (B15).

### Effect of VOCs on *G. smithogilvyi* mycelial growth

The inverted Petri dish assay showed that the VOCs emitted by TRI were the most effective in controlling the mycelial growth of both *G. smithogilvyi* isolates (Fig. 2A and Supplementary Fig. 1). Significant differences (*p* < 0.05) in growth compared with the control were found with TRI treatments at concentrations between 2 × 10^0^ and 2 × 10^1^ (mg/mL) with colony growth of 23.3 ± 0.2 mm (F1N1) and 18.9 ± 0.6 mm (B15) at the concentration 2 × 10^1^ (mg/mL). Both D25 and SUP BCAs did not significantly reduce the mycelial growth of either of the isolates.

### Effect of BCAs on *G. smithogilvyi* growth in dual culture

**Fig. 3:**
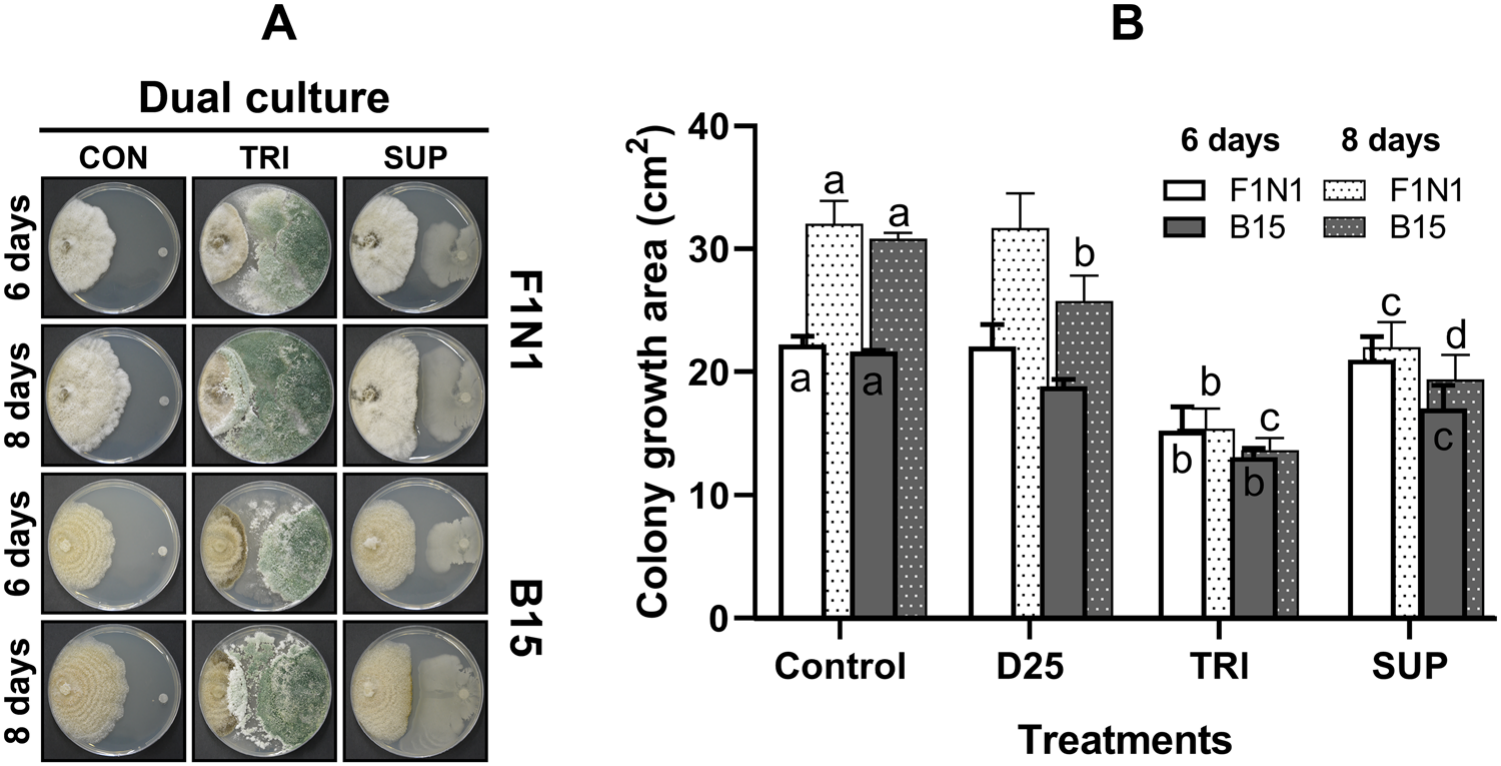
Effect of the biological control agents TRI and SUP on the growth of *G. smithogilvyi* isolates B15 and F1N1 evaluated in a dual culture assay. **A** Inhibition of growth of *G. smithogilvyi* due to exposure to TRI and SUP at 6 and 8 days in the dual culture assay compared with controls. Note for the BCA present in TRI overgrowing *G. smithogilvyi* colony and the halo of inhibition displayed under the SUP treatment. **B** Growth area of the isolates measured after 6 and 8 days. Means ± SEM labelled with different letters are significantly different to the control according to Dunnett’s test at *p* = 0.05

The antagonistic activity of BCAs against *G. smithogilvyi* isolates was assessed in vitro through a dual culture assay on PDA after 6 and 8 days of incubation. Our results showed that the selected BCAs suppressed significantly (*p* < 0.05) the pathogen mycelial growth compared to the control in a time-depended manner (Fig. 3A, B). TRI was the most rapid and effective BCAs in limiting significantly (*p* < 0.05), compared with the controls, the growth of the isolates to 15.2 ± 1.0 and 13.1 ± 0.3 cm^2^ for F1N1 and B15 at 6 days, respectively (Fig. 3B). The intense antagonistic activity of TRI limited the isolates from further growth at 8 days to 15.4 ± 0.9 cm^2^ (F1N1) and 13.6 ± 0.5 cm^2^ (B15). Of particular interest is the apparent impact of TRI on the viability at both 6 and 8 days, *G. smithogilvyi* culture appeared to be killed, as shown by the intense darkening of the culture margin.

Biological control agent SUP was the second most effective at limiting *G. smithogilvyi* colony growth. We observed the earliest effect of SUP on isolate B15 where development was suppressed to 17.0 ± 1.0 cm^2^ at 6 days. In contrast, SUP did not have a significant effect on inhibiting isolate F1N1 colony growth at 6 days. However, SUP was significantly more effective in limiting the growth of both isolates to 22.0 ± 1.1 cm^2^ (F1N1) and 19.3 ± 1.1 cm^2^ (B15) compared to the control after 8 days. On the other hand, D25 was only effective in restricting the growth of isolate B15 to 25.7 ± 1.1 cm^2^ after 8 days compared to the control treatment. The growth area of F1N1 did not differ statistically from the control after being challenged with D25.

### Molecular identification of the isolated biological control agents

Although the commercial products tested were labelled as containing a number of species of *Trichoderma* and/or *Bacillus* and *Pseudomonas*, in the in vitro assays we only found *Trichoderma*-like species (TRI) and *Bacillus*-like species (SUP) to be present. These species were found to be effective in suppressing growth of *G. smithogilvyi* in vitro. Therefore, we further characterised them via Sanger sequencing. The sequencing results showed that TRI yielded sequences that were 100% identical to *T. harzianum* (taxid: 5544) and *T. lignorum* (syn. *viride*) (taxid: 5547) and 98.3% identity to *T. koningii* (taxid: 97,093). On the other hand, SUP yielded sequences with 100% identity to the *Bacillus subtilis* (taxid: 1423). Sequences for *Trichoderma* sp. and *Pseudomonas putida* were not detected in SUP (Supplementary Table 2).

### Identification of nVOCs from solid media through LC–MS

**Fig. 4:**
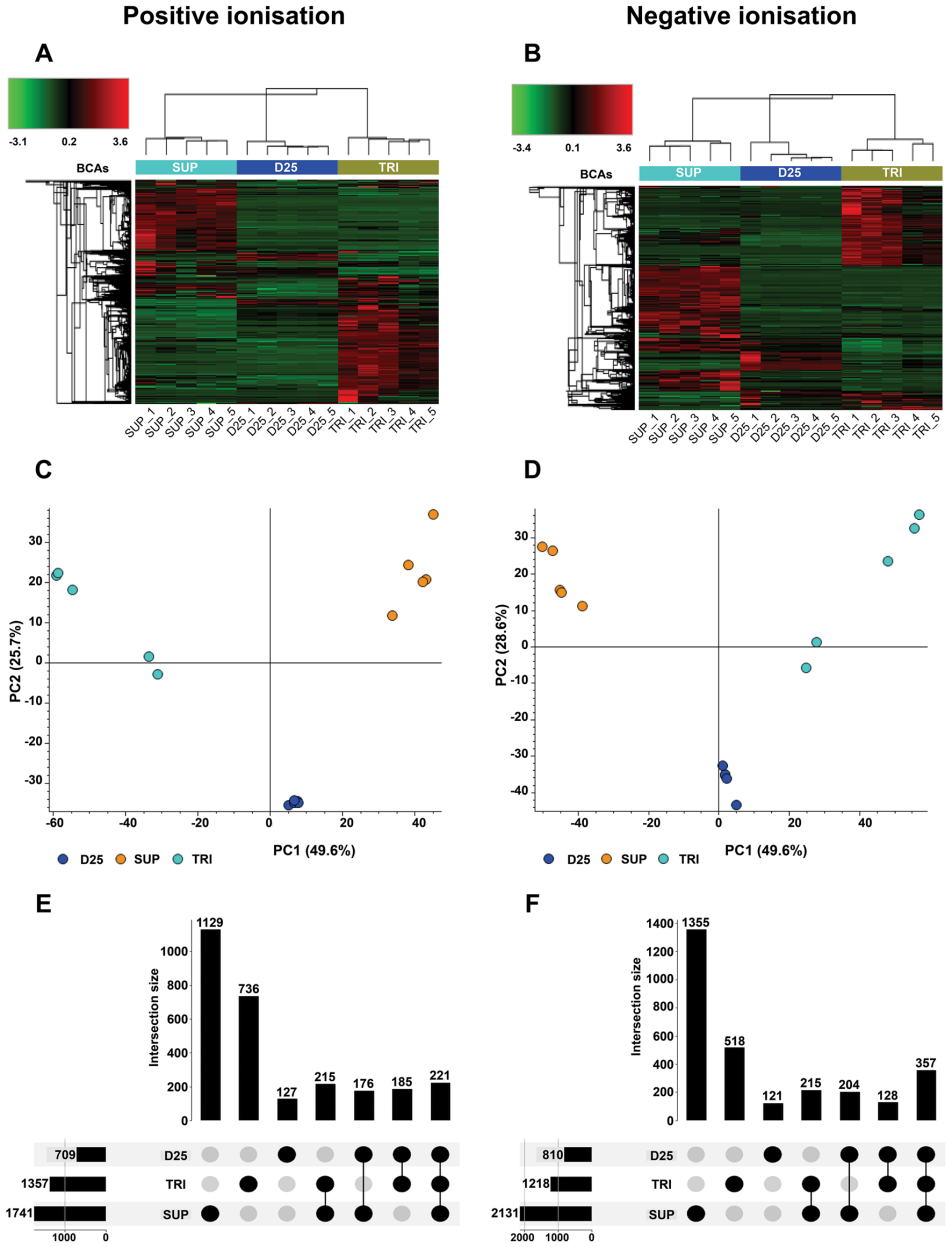
Analysis of the non-volatile compounds (nVOCs) profile of biological control agents SUP, D25 and TRI for the positive (+) and negative (−) ionisation modes. **A**, **B** Heat map analysis, **C**, **D** principal component analysis (PCA) and **E**, **F** UpSet plots for the positive (+) and (−) modes respectively. Heat maps display the normalized compounds abundance in a colour code: low level (green) and high level (red). Both heat maps were clustered with Person’s distance function, and the median was used as the linkage method. UpSet plots show the distribution of nVOCs unique to each BCA (first three bars) and shred between two or three BCAs (last four bars) (Color figure online)

The LC–MS analysis of the positive and negative ionization modes yielded a total of 2789 and 2898 nVOCs respectively. The overall heat maps derived from the Pearson clustering under the positive (Fig. 4A) and negative (Fig. 4C) ionization modes showed the differences in the types of metabolites produced by each BCA. Principal component analysis (PCA) further confirmed the differences between BCAs by clustering the samples in three distinct groups. In the positive ionization mode PCA1 and PCA2 explained 51.3 and 25.7% of the total variance, respectively (Fig. 4C). For the negative ionisation data, PCA1 and PCA2 explained 49.6 and 28.6% of the total variance, respectively (Fig. 4D). Although we used the same amount of crude extract from each BCA for analysis, we observed differences in the total number of metabolites detected. We used an UpSet plot to examine the number of unique and common metabolites across the three BCAs in the positive (Fig. 4E) and negative ionisation modes (Fig. 4F). The majority of nVOCs were molecules present only in SUP. This BCA contributed with 1129 (40.4%) and 1355 (46.7%) to the total number of compounds detected in the positive and negative ion modes respectively. Of the total number of nVOCs detected 26.3% were unique to TRI in the positive ion mode and 17.8% in the negative ion mode. The BCA D25 had the least complex molecular profile with only 4.5% of unique compounds from the total metabolites detected in the positive mode and 4.1% in the negative ionization mode. Moreover, the UpSet plot analysis showed that 221 (7.9%) positively and 357 (12.3%) negatively ionized compounds were commonalities between the three BCAs. On the other hand, about 20% and 18% of the metabolome were commonalities between two of the three BCAs under the positive and negative ionization modes, respectively.

Based on the library search and the FISh scoring assigned to each molecular structure, we were able to putatively annotate 28 nVOCs with a FISh coverage greater than 30 (Table 1). These compounds represented various chemical classes with known bioactivity including aldehydes (one detected), amides (1), aminoalkyl citrates (1), butenolides (1), lipopeptides (4), organic acids (2), peptides (3), polyketides (3), pyrones (3) and terpenes (5). Surprisingly none of these metabolites were shared by the BCAs. Nevertheless, some of the known antimicrobial metabolites detected were for TRI: 6-pentyl-2H-pyran-2-one (C_10_H_14_O_2_) peak at *m/z* = 167.1065 [M + H]^+1^, farnesol (C_15_H_26_O) at *m/z* = 223.2055 [M + H]^+1^, cyclonerodiol (C_15_H_28_O_2_) peak at *m/z* = 241.2162 [M + H]^+1^ and benzoic acid (C_7_H_6_O_2_) with peak at *m/z* = 123.0439 [M + H]^+1^. Some of the featured metabolites detected in SUP were: surfactin B (C_52_H_91_N_7_O_13_) peak *m/z* = 1022.6745 [M + H]^+1^, surfactin C (C_53_H_93_N_7_O_13_) at peak *m/z* = 1036.6899 [M + H]^+1^, cyclo(L-Val-L-Pro) (C_10_H_16_N_2_O_2_) at *m/z* = 197.1284 [M + H]^+1^ and myriocin at *m/z* 400.2849 [M + H]^+1^ (See Supplementary Fig. 2 for myriocin as an example for determination of FISh score). On the other hand, we identified in D25 the following antimicrobial metabolites: 3,7-dimethyloct-6-enal (C_10_H_18_O) peak at *m/z* 137.1324 [M + H–H_2_O]^+1^, 4-hydroxycoumarin (C_9_H_6_O_3_) at *m/z* = 163.0389 [M + H]^+1^, tryptophol (C_10_H_11_NO) peak at *m/z* = 162.0913 [M + H]^+1^, and marinactinone B (C_16_H_26_O_3_) at *m/z* = 267.1955 [M + H]^+1^. The chemical structures of these compounds are shown in Supplementary Fig. 3

### Bioactivity of BCAs crude extracts and assessment of their fractionation

**Fig. 5:**
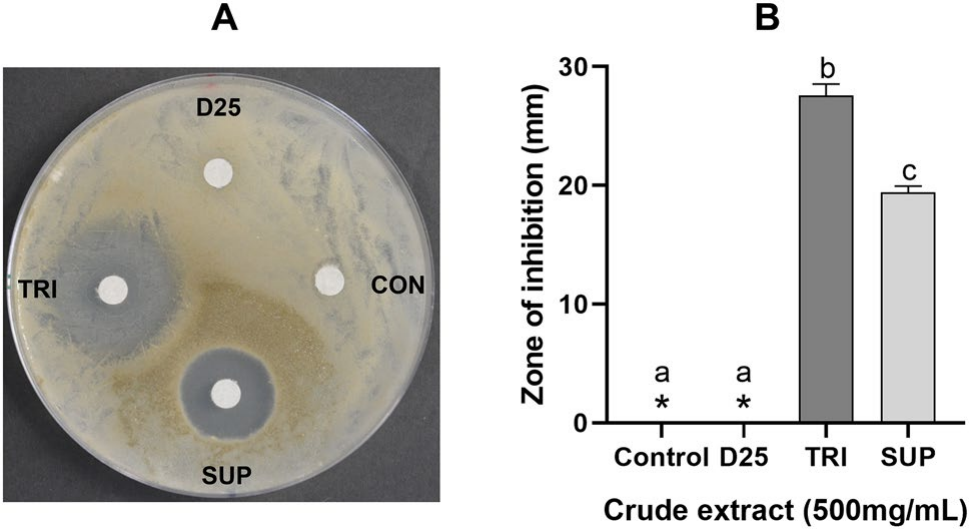
Disc diffusion assay of BCAs methanolic crude extracts (500 mg/mL) and effect on *G. smithogilvyi* conidia germination and mycelial growth. **A** A representative plate displaying the zone of inhibition of each crude extract compared to the control treatment. **B** Size of the zone of inhibition for each BCA. Means ± SEM labelled with different letters are significantly different to the control according to Dunnett’s test at *p* = 0.05. (*) Represents means with a value of zero

Methanolic crude extracts (500 mg/mL) evaluated through the disc diffusion assay showed that TRI and SUP extracts were significantly effective (*p* < 0.05) at inhibiting conidial germination and subsequent mycelial growth of *G. smithogilvyi* isolate B15 (Fig. 5A). These results clearly show the potential effect of nVOCs on suppressing the conidial germination process. In particular, the crude extract obtained from TRI was the most effective inhibitor and displayed a zone of inhibition of 27.6 ± 1.3 mm (Fig. 5B). On the other hand, the methanolic extract derived from SUP was the second most effective and induced a zone of inhibition of 19.6 ± 0.6 mm. In contrast, the crude extract derived from D25 did not show any effect on inhibiting conidia germination and mycelial growth.

The heat map derived from LC–MS data for the positive ionization mode showed that each BCA was distinct in terms of its fractionation profile and that each fraction within a BCA was also quite distinct. All targeted compounds were detected in at least one of the fractions. However, we found that some metabolites were detected in more than one fraction. For example, 5-hydroxyvertinolide was detected in fractions 9 and 10. Similarly, Viridiofungin A was detected in fractions 13 and 14 (Supplementary Fig. 4). This resulted in the fractions having on average 599 (SUP) and 882 (TRI) compounds (Supplementary Fig. 5). However, even though the same amount of fractionated crude extract was applied to each plate, no observable reduction in mycelial growth was found (Supplementary Fig. 6).

## Discussion

The present study provides new information on the evaluation and use of biocontrol-based products to control *G. smithogilvyi* as an alternative for the management of the causal agent of nut rot in chestnuts. The most significant antifungal activity was found for the nVOCs produced by the BCAs. We found that *Trichoderma* species- and *B. subtilis-*derived metabolites suppressed *G. smithogilvyi* mycelial growth and conidial germination. Analysis of nVOCs through LC–MS revealed several antimicrobial compounds from various chemical classes, including aldehydes, aminoalkyl citrates, butenolides, lipopeptides, organic acids, peptides, polyketides, pyrones and terpenes. Our analysis of VOCs showed that only that produced by the formulation TRI were effective in inhibiting pathogen growth. Due to the very limited impact of VOCs on *G. smithogilvyi* and only shown for the BCA TRI under the experimental conditions used here, we have focused on a deeper analysis of nVOCs which, in contrast, showed much stronger bioactivity.

The analysis of TRI-derived nVOCs yielded > 1200 molecules in both ionisation modes, from which we detected eleven known antimicrobial compounds based on their structural FISh scores. For example, the pyrone, 6-pentyl-2H-pyran-2-one (6-PP) has been found to be produced by several *Trichoderma* species, including *T. atroviride* (Garnica-Vergara et al., 2016), *T harzianum* (Rubio et al., 2009), *T. koningii* (Simon et al., 1988) and *T. viride* (Jeleń et al., 2014). This metabolite showed a significant antifungal effect against the ascomycete *Cylindrocarpon destructans*, the causal agent of root rot in *Panax notoginseng* (Chinese ginseng) (Jin et al., 2020). Similarly, evaluation of 6-PP at high concentrations showed significant mycelial growth inhibition of *Fusarium culmorum* in vitro (Jeleń et al., 2014). Another known antimicrobial compound detected in the nVOCs derived from the *Trichoderma* species present in TRI was cyclonerodiol. The effectiveness of cyclonerodiol in controlling fungal pathogens has been demonstrated against the ascomycetes *F. avenaceum* (Wu et al., 2011), *Magnaporthe oryzae* and the oomycete *Phytophthora infestans* (Ngo et al., 2021). Our detection of cyclonerodiol indicates that this antifungal compound may have a role in suppression of *G. smithogilvyi* growth.

The analysis of nVOCs produced by *B. subtilis* present in the product SUP yielded > 1500 metabolites in positive and negative ion modes. Based on the FISh scoring, the most prominent metabolites detected were eleven antimicrobial compounds belonging to various chemical groups including amides, lipopeptides, peptides and polyketides. Within the lipopeptides we identified fengycin and three surfactins (A, B and C). These compounds are cyclic lipopeptides produced by a broad range of *Bacillus* species (Théatre et al., 2021). Studies have shown that these lipopeptides cause cell apoptosis by inducing structural and functional changes in the plasma membrane, cell wall and mitochondrial membrane (Qi et al., 2010; Song et al., 2013). The effectiveness of fengycin and surfactins as antimicrobial compounds has also been shown against a broad range of plant pathogens including bacteria and fungi. For example, Romero et al. (2007) found that fengycin was highly effective at inhibiting conidial germination of *Podosphaera fusca* the causal agent of powdery mildew in cucurbits.

In addition to the above lipopeptides, surfactin A had shown strong inhibition against a number of *Fusarium* species associated with rice bakanae disease (Sarwar et al., 2018). Surfactin B was effective against the bacterium *Xanthomonas oryzae* pv. *oryzae* in vitro (Gun Hee et al., 2011) and surfactin C isomers showed significant inhibition of mycelial growth of *Fusarium oxysporum* f. sp. *lycopersici* and *Aspergillus niger* (Romano et al., 2013). Finally, another relevant metabolite that we detected was the peptide, myriocin, which has been shown to be effective at inhibiting growth of *Fusarium oxysporum* f. sp. *niveum* (Wang et al., 2021) and *F. graminearum* (Shao et al., 2021). Similar to fengycin and surfactins, myriocin acts in the fungal plasma membrane by decreasing its fluidity and destroying its integrity (Wang et al., 2021).

The variation observed in the number of unique metabolites produced by the different BCAs is not uncommon, as their synthesis is influenced by multiple factors. For example, in fungi the type of metabolites depends on the development stage of the species (Calvo et al., 2002) and their number can be increased upon the interaction with other species (Akone et al., 2016; Li et al., 2020). Similarly, the amount and type of metabolites produced in bacteria such as *Bacillus* spp. depends on their growth cycle, with significant increases during the stationary phase (Ayed et al., 2015; Horak et al., 2019). Regardless of the factors that induced the differences in the number of metabolites produced by the BCAs, we have shown that BCAs produced multiple compounds with antifungal properties that are likely to be active against *G. smithogilvyi.* There are almost certainly unknown antimicrobial compounds present in our dataset which could be further investigated through fractionation and application to plates. On the other hand, our HPLC fractionation of crude extract showed that the contribution of individual compounds, such as those discussed above, to the inhibition of fungal growth needs to be further explored. Also, any inhibition is likely due to a combination of compounds or higher concentration of individual metabolites, as some compounds were split over more than one fraction resulting in dilution. Furthermore, the role, if any, of the VOCs secreted by the tested BCAs in suppression of *G. smithogilvyi* growth is still uncertain and could be the subject of further studies either on the tested BCAs or other formulations.

## Conclusion

In conclusion, the present study has revealed the potential of formulated biological control agents to suppress the growth of *G. smithogilvyi* under in vitro conditions. The *Trichoderma* species present in TRI can effectively suppress the pathogen through the secretion of VOCs and nVOCs. In addition, we showed that the *B. subtilis* contained in SUP displayed a strong antifungal activity against the pathogen due to secreted nVOCs alone. Our in vitro studies need now to be expanded into the field to accelerate the implementation of these eco-friendly alternatives for the management of the causal agent of chestnut rot.

**Table 1:** Non-volatile compounds (nVOCs) produced by formulated BCAs D25, SUP and TRI detected by LC–MS

Tentative compound identification	Molecular formula	Cal. Mol.^a^ mass	m/z	Δ mass (ppm)	FISh coverage	Ion	RT (min)	D25	SUP	TRI	Activity	References
Aldehyde												
3,7-dimethyloct-6-enal	C_10_H_18_O	154.1357	137.1324	− 0.27	80	[M + H-H_2_O]^+1^	6.4	✓^b^	–	–	Antibacterial	Singh et al. (2018)
Amides												
Bacillamidin B	C_19_H_35_NO_5_	357.2515	358.2588	0.0	42	[M + H]^+1^	7.0	–	✓	–	Antibacterial	Zhou et al. (2018)
Aminoalkyl citrates												
Viridiofungin A	C_31_H_45_NO_10_	591.3040	592.3113	− 0.53	76	[M + H]^+1^	7.8	–	–	✓	Antifungal	El-Hasan et al. (2009)
Butenolides												
5-hydroxyvertinolide	C_14_H_18_O_5_	266.1154	289.1046	0.16	66	[M + Na]^+1^	6.0	–	–	✓	Antifungal	Derntl et al. (2017)
Lipopeptides												
Fengycin	C_72_H_110_N_12_O_20_	1462.7953	732.4049	− 0.38	33	[M + 2H]^+2^	6.9	–	✓	–	Antifungal	Romero et al. (2007)
Surfactin A	C_51_H_89_N_7_O_13_	1007.6515	1008.6589	− 026	43	[M + H]^+1^	10.7	–	✓	–	Antifungal	Sarwar et al. (2018)
Surfactin B	C_52_H_91_N_7_O_13_	1021.6671	1022.6745	− 0.29	85	[M + H]^+1^	11.1	–	✓	–	Antibacterial	Gun Hee et al. (2011)
Surfactin C	C_53_H_93_N_7_O_13_	1035.6826	1036.6899	− 0.45	56	[M + H]^+1^	11.8		✓		Antifungal	Aleti et al. (2016)
Organic acids												
2-methylbutanoic acid	C_5_H_10_O_2_	102.0681	101.0608	0.16	38	[M-H]^−1^	1.2	✓	–	–	Antibacterial	Hayashida-Soiza et al. (2008)
Benzoic acid	C_7_H_6_O_2_	122.0367	123.0439	− 0.55	80	[M + H]^+1^	4.7			✓	Antifungal	Nehela et al. (2021)
Peptides												
Bacilysin	C_12_H_18_N_2_O_5_	270.1215	269.1143	0.08	47	[M-H]^−1^	0.9	–	✓	–	Antibacterial	Wu et al. (2015)
Cyclo(L-Val-L-Pro)	C_10_H_16_N_2_O_2_	196.1211	197.1284	− 0.08	61	[M + H]^+1^	3.9	–	✓	–	Antibacterial	Zin et al. (2020)
Myriocin	C_21_H_39_NO_6_	401.2777	400.2849	− 0.06	72	[M + H]^+1^	8.8	–	✓	–	Antifungal	Wang et al. (2021)
Polyketides												
Macrolactin A	C_24_H_34_O_5_	402.2407	403.2479	0.26	35	[M + H]^+1^	5.2	–	✓	–	Antibacterial	Chen et al. (2019)
Macrolactin-O	C_30_H_44_O_10_	564.2937	609.2919	0.56	53	[M + FA-H]^−1^	6.0	–	✓	–	Antibacterial	Zheng et al. (2007)
Oxydifficidin	C_31_H_45_O_7_P	560.2901	1143.5695	− 0.34	30	[2 M + Na]^+1^	8.6	–	✓	–	Antibacterial	Im et al. (2020)
Pyrones												
6-pentyl-2H-pyran-2-one	C_10_H_14_O_2_	166.0993	167.1065	− 037	87	[M + H]^+1^	7.2	–	–	✓	Antifungal	Jin et al. (2020)
Viridepyronone	C_10_H_12_O_3_	180.0786	181.0858	− 0.20	76	[M + H]^+1^	5.0	–	–	✓	antifungal	Evidente et al. (2003)
Marinactinone B	C_16_H_26_O_3_	266.1882	267.1955	0.17	60	[M + H]^+1^	8.3	✓	–	–	Cytotoxic	Wang et al. (2011)
Terpenes												
Cyclonerodiol	C_15_H_28_O_2_	240.2089	241.2162	0.04	83	[M + H]^+1^	6.1	–	–	✓	Antifungal	Wu et al. (2011)
Farnesol	C_15_H_26_O	222.1982	223.2055	− 0.37	87	[M + H]^+1^	7.1	–	–	✓	Antifungal	Cotoras et al. (2013)
Trichocarane A	C_15_H_26_O_3_	254.1881	255.1954	− 0.14	50	[M + H]^+1^	6.0	–	–	✓	G. inhibitor	Macías et al. (2000)
Trichocarotin C	C_15_H_22_O_3_	250.1569	251.1642	0.06	75	[M + H]^+1^	6.0	–	–	✓	Antimicroalgal	Shi et al. (2018)
Trichocitrin	C_20_H_28_O	284.2140	258.2213	0.08	33	[M + H]^+1^	9.8	–	–	✓	Antibacterial	Liang et al. (2016)
Others												
4-hydroxycoumarin	C_9_H_6_O_3_	162.0316	163.0389	− 0.43	66	[M + H]^+1^	6.2	✓	–	–	Antibacterial	Yang et al. (2016)
Formononetin	C_16_ H_12_O_4_	268.0736	291.0628	0.16	33	[M + Na]^+1^	6.2	✓	–	–	Antifungal	das Neves et al. (2016)
Indirubin	C_16_H_10_N_2_O_2_	262.0742	263.0815	0.08	50	[M + H]^+1^	4.4	–	–	✓	Antifungal	Ponnusamy et al. (2010)
Tryptophol	C_10_H_11_NO	161.0840	162.0913	− 0.19	62	[M + H]^+1^	5.2	✓	–	–	Antifungal	Singkum et al. (2019)

^b^() compound present, (–) compound absent

^a^Calculated molecular mass

## Supplementary Information

Below is the link to the electronic supplementary material.

Supplementary file1 (PDF 327 KB)

Supplementary file2 (PDF 291 KB)

Supplementary file3 (PDF 262 KB)

Supplementary file4 (PDF 285 KB)

Supplementary file5 (PDF 440 KB)

Supplementary file6 (PDF 499 KB)

Supplementary file7 (DOCX 16 KB)

Supplementary file8 (DOCX 16 KB)

## Data Availability

The datasets generated during and/or analysed during the current study are not publicly available due to commercial confidentiality but are available from the corresponding author on reasonable request.
