# Supplementary material for: Metabolites derived from fungi and bacteria suppress in vitro growth of Gnomoniopsis smithogilvyi, a major threat to the global chestnut industry
Source: Metabolomics. 2022 Sep 15;18(9):74. doi: 10.1007/s11306-022-01933-4 (PMC9474450; doi:10.1007/s11306-022-01933-4)
Supplement: Supplementary file 1 — Supplementary file1 (PDF 327 KB) [file 11306_2022_1933_MOESM1_ESM.pdf]

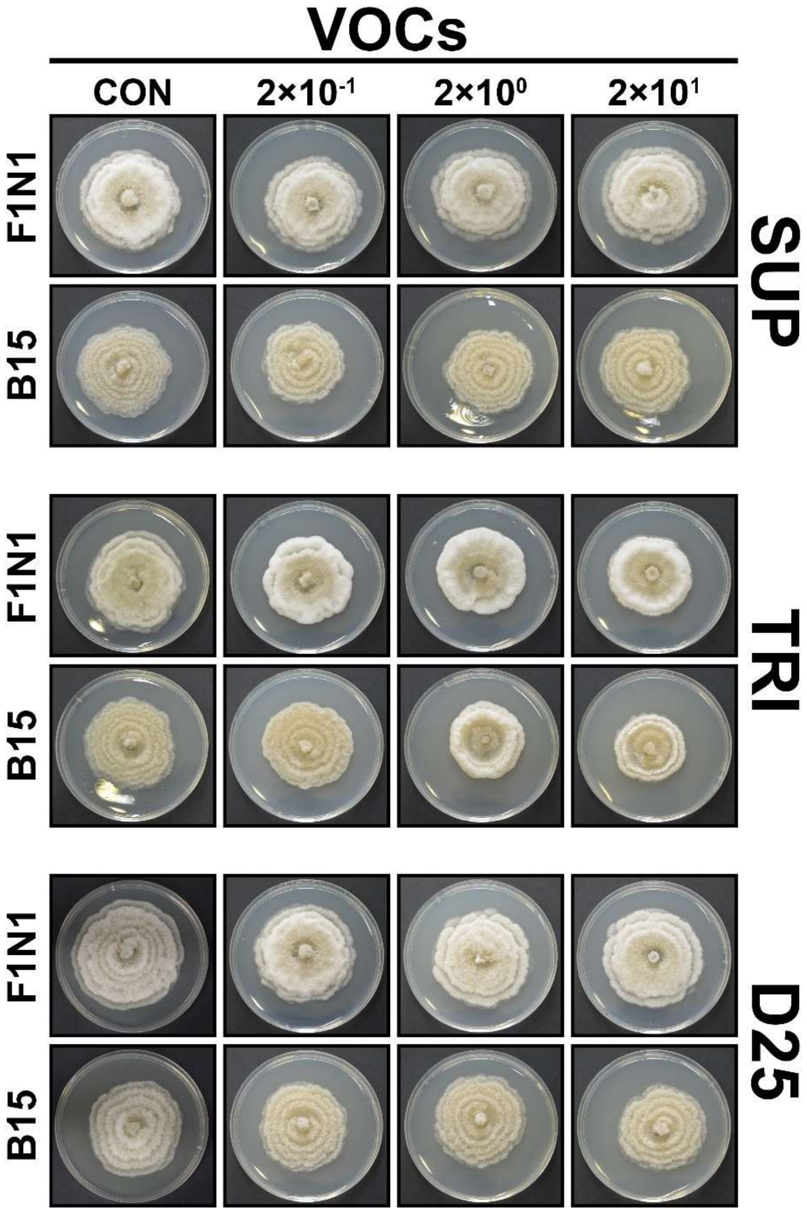

Supplementary Fig. 1 Representation of the effect of VOCs emitted by the highest three BCA (SUP, TRI, D25) concentrations (µg/mL) tested on the mycelial growth of *G. smithogilvyi* isolates F1N1 and B15. Plates were incubated at 23 °C in the dark for six days. For statistical analysis refer to Figure 2A.
