# Supplementary material for: Metabolites derived from fungi and bacteria suppress in vitro growth of Gnomoniopsis smithogilvyi, a major threat to the global chestnut industry
Source: Metabolomics. 2022 Sep 15;18(9):74. doi: 10.1007/s11306-022-01933-4 (PMC9474450; doi:10.1007/s11306-022-01933-4)
Supplement: Supplementary file 2 — Supplementary file2 (PDF 291 KB) [file 11306_2022_1933_MOESM2_ESM.pdf]

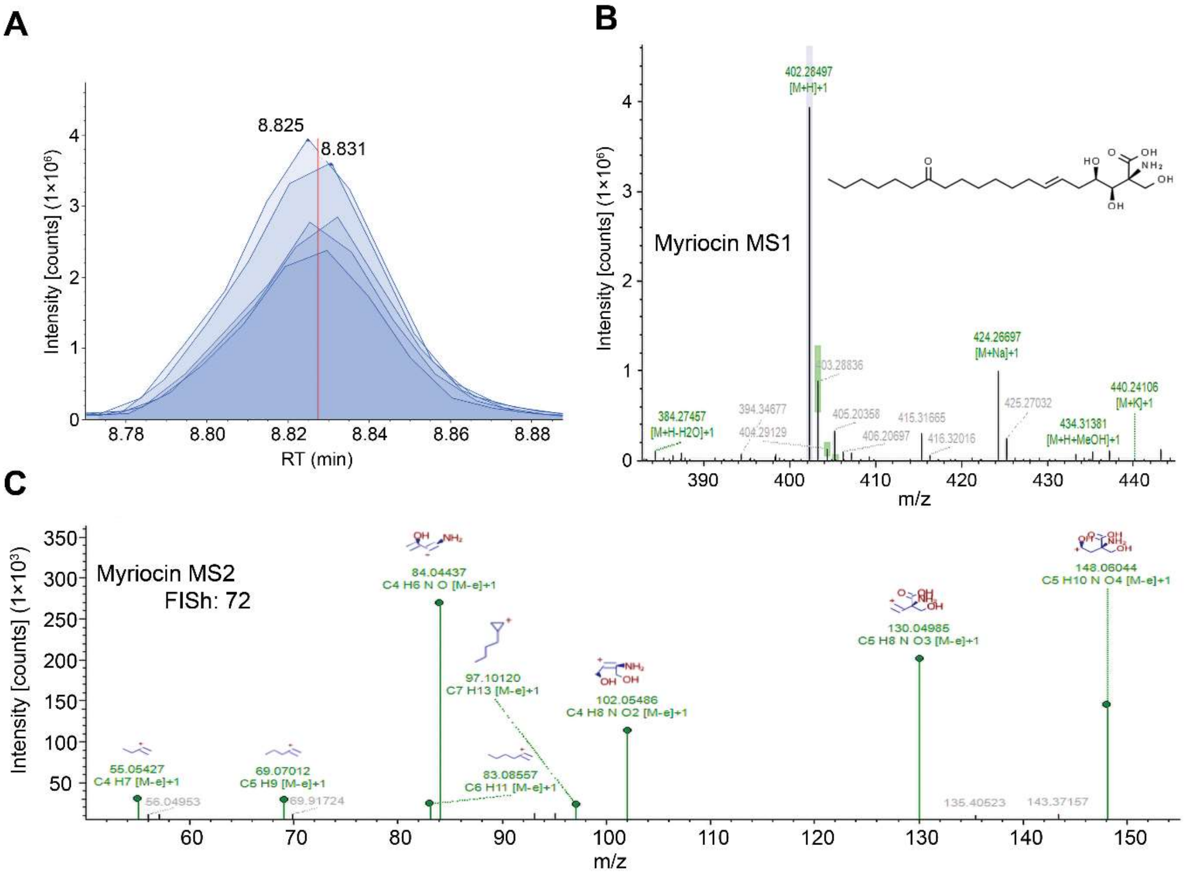

Supplementary Fig. 2 Representation of the annotation process of non-volatile compounds (nVOCs) using myriocin as an example. (A) Overlay of five technical replicates of ion chromatograms for myriocin from *G. smithogilvyi* extracts. (B) Full scan spectrum (MS1) showing the parent ion at 402.28497 m/z. (C) Product ion spectrum (MS2) for myriocin with the annotated fragments determined using the FISh algorithm:72.
