# Supplementary material for: Metabolites derived from fungi and bacteria suppress in vitro growth of Gnomoniopsis smithogilvyi, a major threat to the global chestnut industry
Source: Metabolomics. 2022 Sep 15;18(9):74. doi: 10.1007/s11306-022-01933-4 (PMC9474450; doi:10.1007/s11306-022-01933-4)
Supplement: Supplementary file 3 — Supplementary file3 (PDF 262 KB) [file 11306_2022_1933_MOESM3_ESM.pdf]

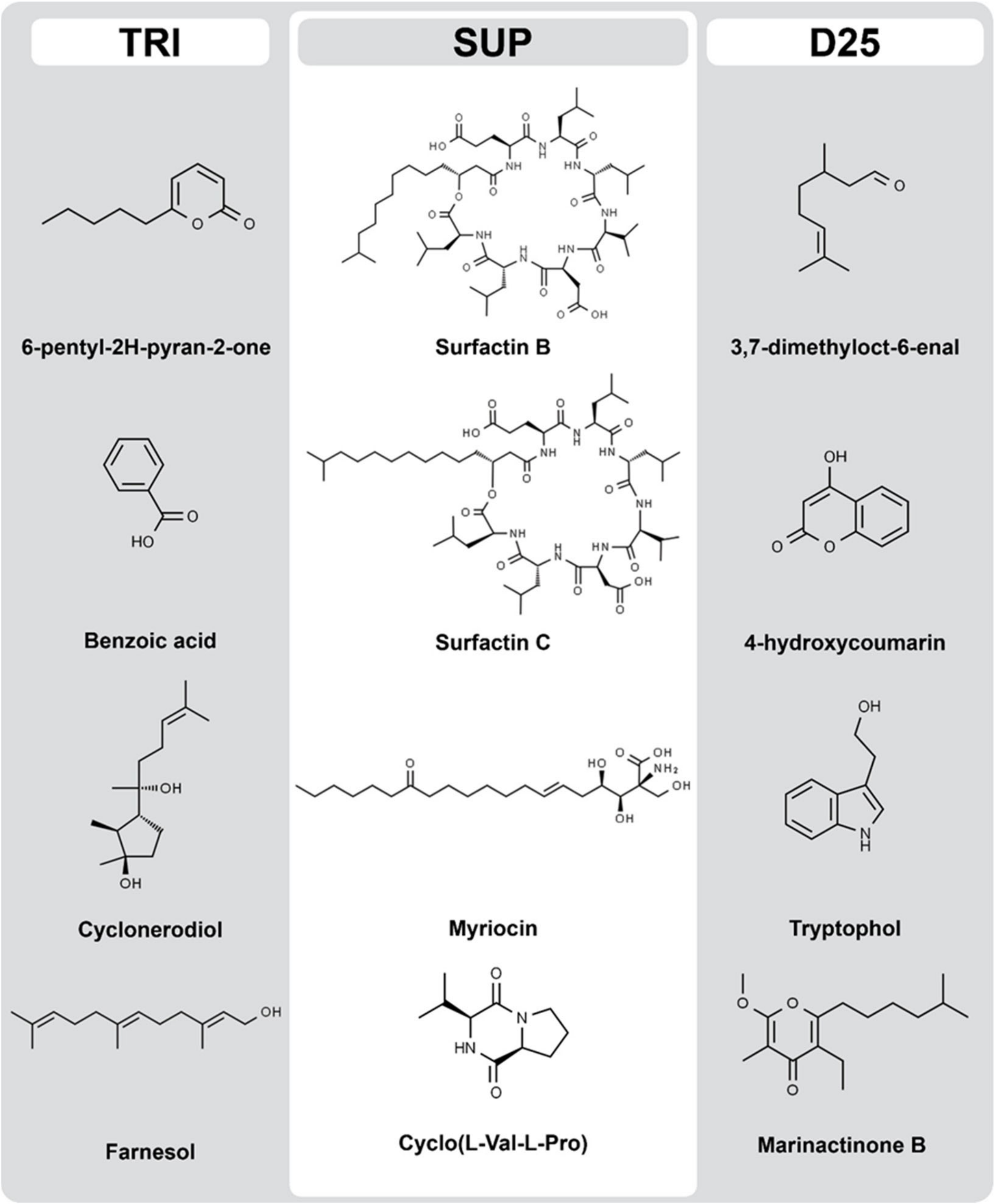

Supplementary Fig. 3 Chemical structures of high FISh scoring non-volatile compounds (nVOCs) detected in the methanolic extract of the biological control agents TRI, SUP and D25.
