# Supplementary material for: Metabolites derived from fungi and bacteria suppress in vitro growth of Gnomoniopsis smithogilvyi, a major threat to the global chestnut industry
Source: Metabolomics. 2022 Sep 15;18(9):74. doi: 10.1007/s11306-022-01933-4 (PMC9474450; doi:10.1007/s11306-022-01933-4)
Supplement: Supplementary file 4 — Supplementary file4 (PDF 285 KB) [file 11306_2022_1933_MOESM4_ESM.pdf]

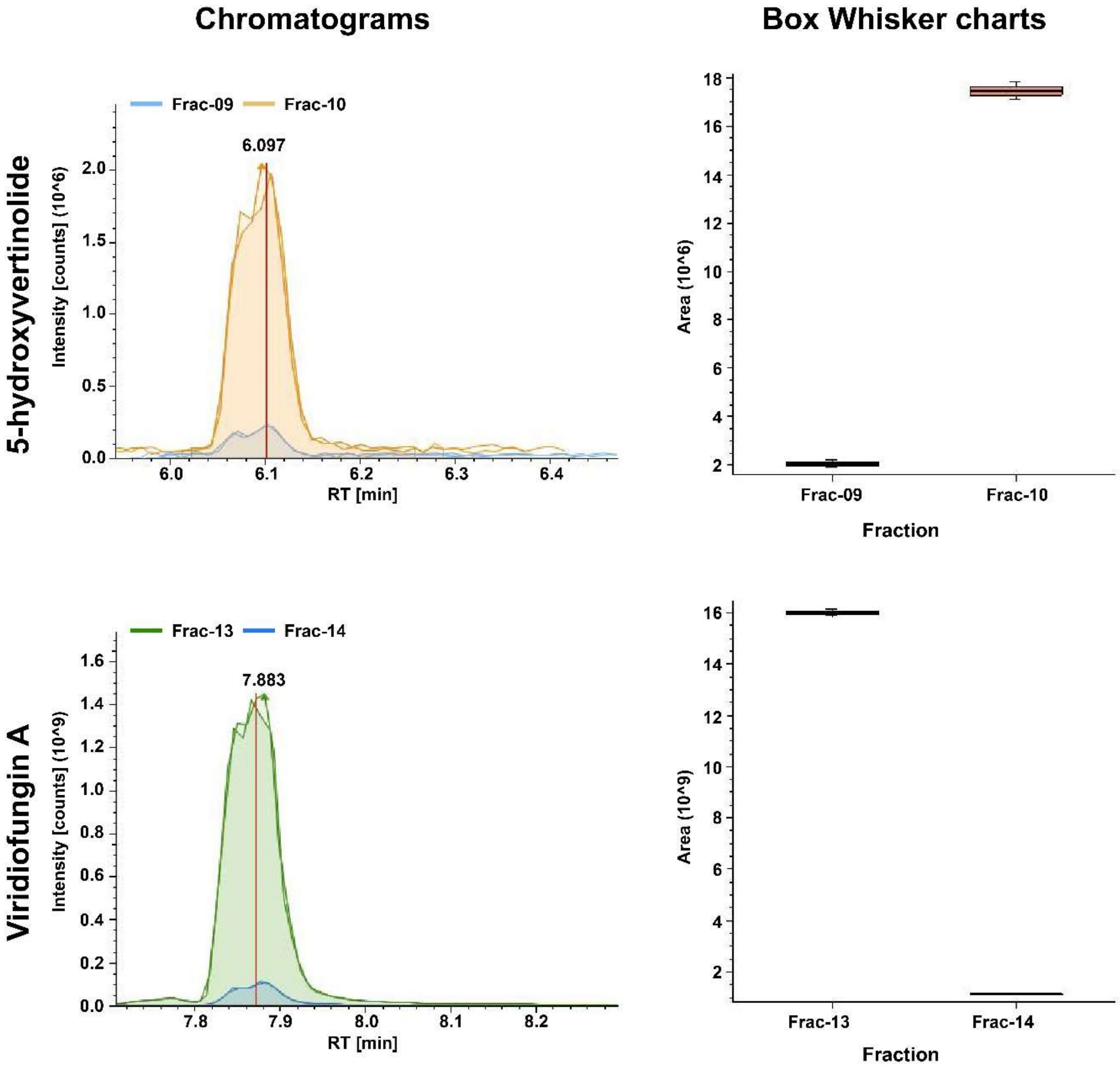

Supplementary Fig. 4 Example of metabolites detected in more than one fraction. Chromatograms show the intensity of the peaks and retention time (min) for each compound in each fraction (Frac). Box Whisker charts show the relative abundance of each compound in each fraction.
