# Supplementary material for: Metabolites derived from fungi and bacteria suppress in vitro growth of Gnomoniopsis smithogilvyi, a major threat to the global chestnut industry
Source: Metabolomics. 2022 Sep 15;18(9):74. doi: 10.1007/s11306-022-01933-4 (PMC9474450; doi:10.1007/s11306-022-01933-4)
Supplement: Supplementary file 5 — Supplementary file5 (PDF 440 KB) [file 11306_2022_1933_MOESM5_ESM.pdf]

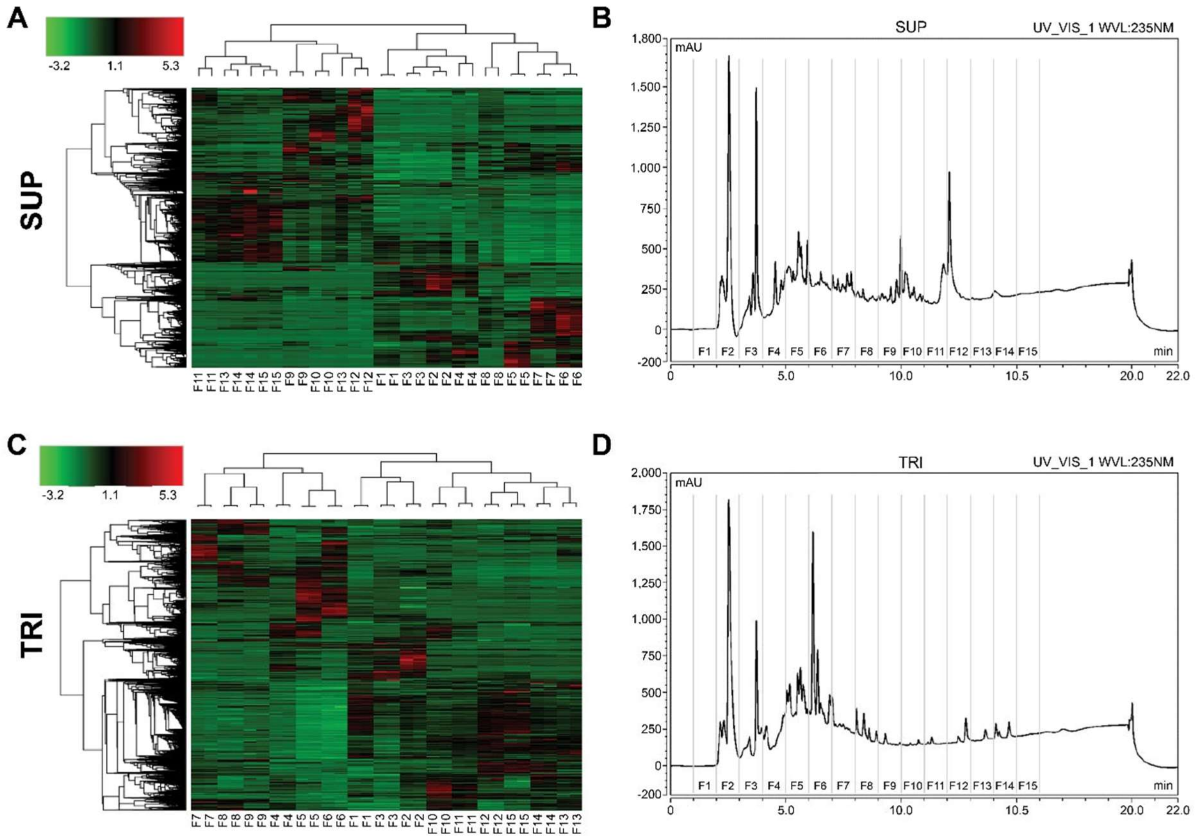

Supplementary Fig. 5 Analysis of non-volatile compounds (nVOCs) profiles from HPLC fractions derived from the biological control agents SUP and TRI. (**A**) Heat map analysis and (**B**) HPLC chromatogram of SUP fractions. (**C**) and (**D**) Heat map and chromatogram of TRI. Both chromatograms were obtained at 235 nm. Heat maps display the normalized compound abundance (positive mode) in a colour code: low level abundance (green) and high-level abundance (red). Both heat maps were clustered with Person's distance function, and the median was used as the linkage method.
