# Supplementary material for: Metabolites derived from fungi and bacteria suppress in vitro growth of Gnomoniopsis smithogilvyi, a major threat to the global chestnut industry
Source: Metabolomics. 2022 Sep 15;18(9):74. doi: 10.1007/s11306-022-01933-4 (PMC9474450; doi:10.1007/s11306-022-01933-4)
Supplement: Supplementary file 6 — Supplementary file6 (PDF 499 KB) [file 11306_2022_1933_MOESM6_ESM.pdf]

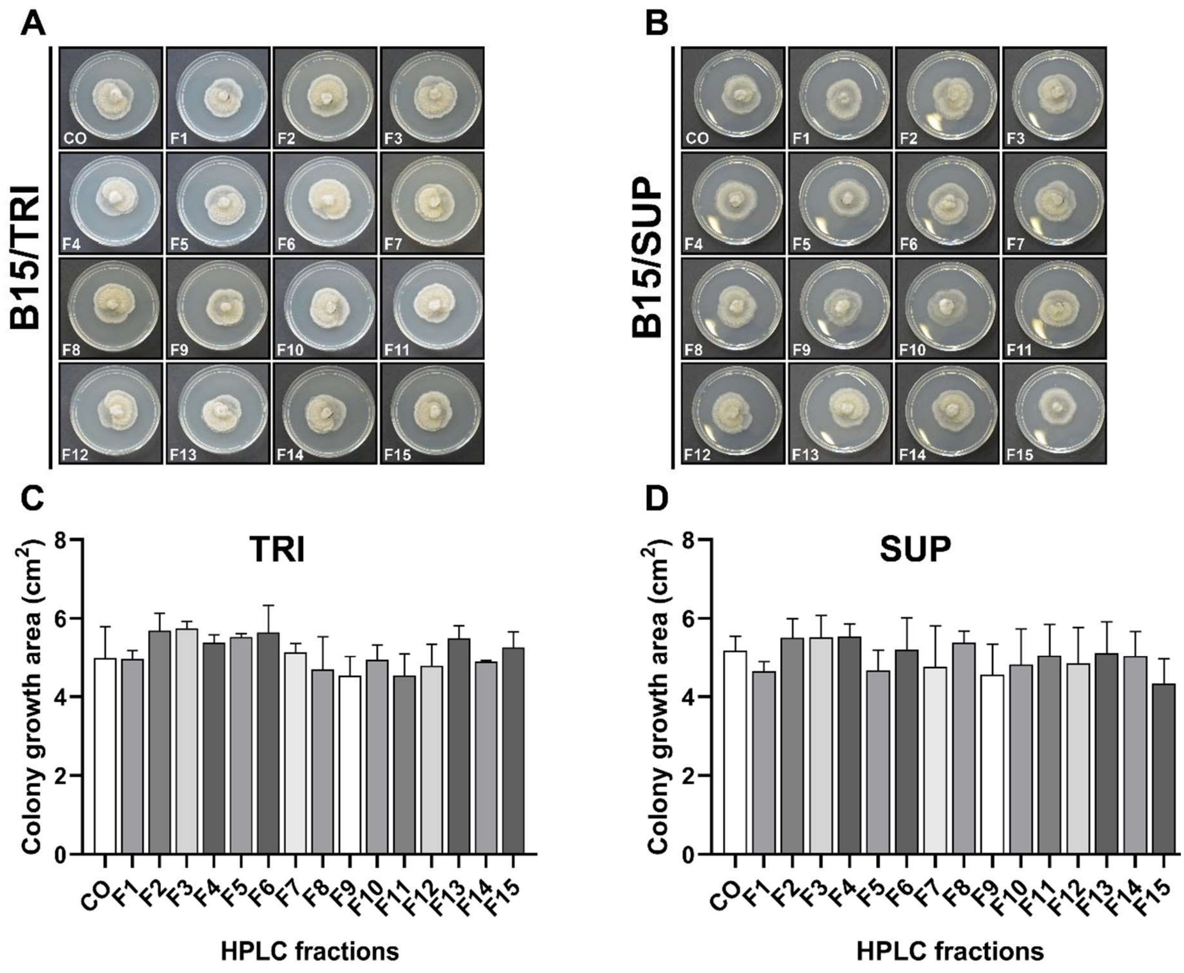

Supplementary Fig. 6 Effect of HPLC fractions derived from the biological control agents TRI and SUP on *G. smithogilvyi* isolate B15 mycelial growth. Effect of the BCA TRI (**A**) and SUP (**B**) on mycelial growth. Plates were incubated at 23 °C in the dark for three days. Colony area of isolate B15 under treatment with TRI (**C**) and SUP (**D**). Means ± SEM were not significantly different to the control according to Dunnett's test at $p = 0.05$.
