# Supplementary material for: Metabolites derived from fungi and bacteria suppress in vitro growth of Gnomoniopsis smithogilvyi, a major threat to the global chestnut industry
Source: Metabolomics. 2022 Sep 15;18(9):74. doi: 10.1007/s11306-022-01933-4 (PMC9474450; doi:10.1007/s11306-022-01933-4)
Supplement: Supplementary file 7 — Supplementary file7 (DOCX 16 KB) [file 11306_2022_1933_MOESM7_ESM.docx]

| **Supplementary Table 1** Composition and concentration used of the commercial BCAs tested | | | | |
| --- | --- | --- | --- | --- |
| **Commercial name** | **Acronym** | **Manufacturer** | **Composition** | **Concentration of BCA** |
| Nutri-Life Tricho-Shield™ | TRI | Nutri-Tech Solutions | *Trichoderma harzianum, T. koningii, T. lignorum* (syn. v*iride*) | 0.002- 20 (mg/mL) |
| Superzyme® | SUP | JH Biotech | *Bacillus subtilis, Pseudomonas putida, T. harzianum, T. koningii* | 0.002- 20 (mg/mL) |
| TRI-D25 | D25 | JH Biotech | *T. harzianum, T. koningii* | 0.002- 20 (μL/mL) |
|  | | | | |
