# Supplementary material for: Metabolites derived from fungi and bacteria suppress in vitro growth of Gnomoniopsis smithogilvyi, a major threat to the global chestnut industry
Source: Metabolomics. 2022 Sep 15;18(9):74. doi: 10.1007/s11306-022-01933-4 (PMC9474450; doi:10.1007/s11306-022-01933-4)
Supplement: Supplementary file 8 — Supplementary file8 (DOCX 16 KB) [file 11306_2022_1933_MOESM8_ESM.docx]

| **Supplementary Table 2.** Molecular identification of microbial components of TRI and SUP | | | | | | |
| --- | --- | --- | --- | --- | --- | --- |
| **Product** | **Species** | **Total score** | **Query cover (%)** | **E-value** | **Identity** | **Accession^a^** |
| TRI | *T. harzianum* | 444 | 100 | 1⨯10‐124 | 100 | MH127469.1 |
|  | *T. lignorum* (syn. v*iride*) | 444 | 100 | 1⨯10‐124 | 100 | MH333256.1 |
|  | *T. koningii* | 422 | 100 | 5⨯10‐118 | 98.34 | KY305047.1 |
| SUP | *T. harzianum* | – | – | – | – | – |
|  | *T. koningii* | – | – | – | – | – |
|  | *Bacillus subtilis* | 444 | 100 | 5⨯10‐123 | 100 | MT623577.1 |
|  | *Pseudomonas putida* | – | – | – | – | – |
| (–) DNA from the respective species was not detected through amplification of the ITS or 16S regions  a Accession number corresponds to the sequences used to compare BCA amplicons available in NCBI data base. | | | | | | |
